# Risk Perception and Knowledge, Attitudes, and Practices Against COVID-19 in a Hypertensive Population From a Semi-Urban City of Ecuador

**DOI:** 10.3389/fpubh.2021.734065

**Published:** 2021-12-14

**Authors:** Teresa Aumala, Maria Cardenas, Daniel Vergara, Monserrate Vasconez, Ivan Palacios, Enrique Terán

**Affiliations:** ^1^Ministerio de Salud Publica del Ecuador, Quito, Ecuador; ^2^Colegio de Ciencias de la Salud, Universidad San Francisco de Quito, Quito, Ecuador

**Keywords:** COVID-19, SARS-CoV-2 pandemic, hypertension, elderly people, risk perception and knowledge

## Abstract

**Background:** In an elderly population with hypertension, severe acute respiratory syndrome coronavirus 2 (SARS-CoV-2) is associated with a higher incidence of mortality and a protracted course of clinical symptoms.

**Objective:** To assess the perceived risk of infection and complications due to COVID-19 in people with hypertension living in a semi-urban city of Ecuador.

**Methods:** A cross-sectional telephone survey of adult outpatients with a previous diagnosis of hypertension in the semi-urban community of Conocoto in Quito, Ecuador was conducted from August to December 2020.

**Results:** A total of 260 adult outpatients, aged 34–97 years, completed telephone surveys. Of total, 71.5% (*n* = 186) of respondents were women and 28.5% (*n* = 74) of respondents were men. Overall, 18.1% believe that their risk of infection is “very high,” 55.4% believe that their risk of infection is “high,” 21.5% believe that their risk of infection is “low,” and 5% believe that their risk of infection is “very low.” The perceived risk of complications, if infected by COVID-19, revealed that 21.9% believe that their risk of complication is “very high,” 65.0% believe that their risk of complication is “high,” 10.4% believe that their risk of complication is “low,” and 2.7% believe that their risk of complication is “very low.”

**Conclusion:** Patients with hypertension are aware of the risks posed by COVID-19 infection and its impact on their health. However, the health system must educate the population on health practices and behaviors to avoid COVID-19 infection until the majority of the population of Ecuador can be vaccinated.

## Introduction

The severe acute respiratory syndrome coronavirus 2 (SARS-CoV-2) pandemic, also known as COVID-19, has had a serious impact on both physical and mental health globally. Around 110 million cases and 2 million deaths have been recorded worldwide (1, 2). As of February 17, 2021, Ecuador has registered 270,000 cases of COVID-19, with the majority of cases in the provinces of Guayas and Pichincha. Also up to this date, 15,400 deaths have been registered in the country (3, 4).

Due to the COVID-19 pandemic, the city of Quito and its neighboring towns had been in a so-called “*red light”* since mid-March 2020, which meant the lockdown of the entire population. Almost 4 months later, on July 1, 2020, the alert status went from red to yellow and then to limited mobility according to vehicle license plate number, maximum capacities of 30% in commercial establishments, and a curfew from 11 p.m. to 5 a.m. (5).

Conocoto is one of the largest semi-urban towns in Ecuador, located 8 km south of Quito, with a population of 100,072 and it was one of the sites most affected during the COVID-19 pandemic. COVID-19 cases jumped from 60 in April 2020 to 3,548 in February 2021, mainly attributed to the increase in the freedom afforded by the change in alert status (3, 6).

At the same time, high blood pressure is one of the most common chronic noncommunicable diseases of the world, affecting 1 out of 4 men and 1 out of 5 women (7) and in Ecuador, the prevalence of hypertension is 9.3% according to the latest official data (8). Moreover, although the entire population is susceptible to COVID-19 infection, people with chronic nontransmissible diseases such as hypertension (9) are more vulnerable, have more severe symptoms, and suffer more serious complications than the general population (10). People with hypertension are 2.5 times more likely to become infected by COVID-19 (11) and 4 times more likely to have a more severe clinical presentation than the general population (12). Also, their risk of dying from COVID-19 increases 2.5-fold compared to the general population (11).

A study conducted in Peru, for example, found the most common comorbidities associated with mortality due to COVID-19 were obesity, hypertension, and diabetes mellitus (13), while in Malaysia or India, most of the deaths due to COVID-19 occurred in individuals with diabetes and hypertension, and in those aged 70 years or older (14, 15).

However, little is known about the perceived risk of COVID-19 infection among semi-urban dwelling adult outpatients with confirmed hypertension or about the efficacy of current protocols for avoiding COVID-19 infection.

Thus, the objective of this study is to investigate the knowledge, attitudes, and practices to combat COVID-19 and the self-perception of risk in a population with hypertension from a semi-urban city in Ecuador.

## Materials and Methods

This was an observational and cross-sectional study in the health center of Conocoto, Ecuador. This health center has a database of 734 hypertensive patients who periodically come for treatment.

The sample size was 260 participants calculated using a 5% error rate and a power of 80%. This study was approved by the Bioethics Committee at Universidad San Francisco de Quito (2020-047M).

Participants were randomly selected from the database and received a telephone survey conducted between August and December 2020. The survey included information concerning age, gender, management of arterial hypertension, comorbidities, management of comorbidities, symptoms, self-perceived risk of contagion, experience with COVID-19 testing, knowledge, and practices to combat infection by COVID-19 such as handwashing and mask use.

Inclusion criteria included adults aged 18 years and older with a prior history of hypertension and agreement to voluntary and anonymous participation. Surveys with incomplete data or those that had been completed incorrectly were excluded from this study.

All the data collected were recorded and organized into a spreadsheet for later processing with Jamovi (version 1.6) for the respective statistical analysis. A descriptive analysis of the sample was carried out, where the percentages were obtained for characterization of the sample and description of the variables evaluated in the survey. Inferential analysis between men and women was based on Fisher's exact test for qualitative data and the Student's *t*-test for quantitative results.

We organized our analysis into three parts. First, we present the epidemiological description of the population. Then, we present the self-perceived risk of infection and complications in the hypertensive population. Finally, we present the perceptions of the various prevention mechanisms and attitudes with respect to health practices during the COVID-19 pandemic.

## Results

From the 384 hypertensive subjects selected and contacted by phone, 69.8% (*n* = 268) of hypertensive subjects agreed to participate in this study, and data from 8 subjects were eliminated due to inconsistencies. Thus, data from a total of 260 patients were used for analysis.

The mean age of the participants was 64.5 ± 13.6 years (in the age range 34–97 years) and most of the participants were women (Table 1). Most of the survey respondents were already retired or working at home. All the subjects self-identified as hypertensive, but only 83% (*n* = 217) of subjects mentioned receiving any treatment, with no differences between men and women (Table 1). However, 8.8% (*n* = 19) of subjects mentioned that despite treatment, their hypertension was not under control. This was significantly more common in women (Table 1). No information about the treatment itself was retrieved. Also, among comorbidities, central nervous system disorders, including depression, dementia, stroke, or Parkinson's disease, were most prevalent in both groups, followed by obesity and thyroid disease, among others (Table 1). However, 1 out of every 4 participants, independent of gender, had 2 or more comorbidities.

**Table 1:** Characteristics of the hypertensive subjects from a semi-urban city of Ecuador.

	**Men**	**Women**	**p value**
	**(*n* = 74, 28.5%)**	**(*n* = 186, 71.5%)**	
Age (years)	64.5 ± 12.7	62.4 ± 14.0	0.2638
Receiving treatment for HBP	57 (77.0%)	160 (86.0%)	0.096
Controlled HBP (self-perception)	54 (94.7%)	144 (77.4%)	0.0029
Comorbidities			
None	25 (33.8%)	45 (24.2%)	0.1236
Diabetes	2 (2.7%)	16 (8.6%)	0.1087
Cancer	3 (4.1%)	5 (2.7%)	0.6918
COPD	2 (2.7%)	5 (2.7%)	1.0000
Renal disease	2 (2.7%)	6 (3.2%)	1.0000
Obesity	7 (9.5%)	13 (7.0%)	0.6061
Thyroid disease	3 (4.1%)	13 (7.0%)	0.5683
Cardiac disease	2 (2.7%)	5 (2.7%)	1.0000
CNS disease	10 (13.5%)	28 (15.1%)	0.8472
Two or more	18 (24.3%)	50 (26.9%)	0.7553
Comorbidity with treatment (yes)	34 (69.4%)	113 (80.1%)	0.1643
Comorbidity under control (yes)	30 (88.2%)	109 (96.5%)	0.0837

*HBP, high blood pressure; COPD, chronic obstructive pulmonary disease; CNS, central nervous system*.

With respect to COVID-19, all the subjects knew that it is caused by SARS-CoV-2, but 38.5% (*n* = 100) of the participants believed that it was the same as the common flu. However, only 73.5% (*n* = 191) of the participants perceived themselves as being at high risk for infection by COVID-19 soon, i.e., in the following 2 or 3 months. A higher proportion of men endorsed a lower perceived risk (see Table 2). Moreover, only 38.8% (*n* = 101) of the participants self-perceived to be at a higher risk for COVID-19 infection compared to the general population, but in this case, there were no differences between groups (see Table 2).

**Table 2:** Perceived risk of coronavirus disease 2019 (COVID-19) among semi-urban dwelling adults with hypertension.

	**High**		**Same**		**Low**	
	**Men**	**Women**	**Men**	**Women**	**Men**	**Women**
Risk of COVID-19 infection	47 (63.5)	144 (77.4)			27 (36.5)	42 (22.6)
	*p* = 0.03				
Risk of COVID-19 infection vs. general population	25 (33.8)	76 (40.9)	30 (40.5)	82 (44.1)	19 (25.7)	28 (15.1)
	*p* = 0.32				
Risk of complications due to COVID-19 infection	64 (86.5)	162 (87.1)			10 (13.5)	24 (12.9)
	*p* = 1.00				
Risk of complications due to COVID-19 infection vs. general population	34 (46.0)	91 (48.9)	31 (41.9)	80 (43.0)	9 (12.2)	15 (8.1)
	*p* = 0.68				

There was also no difference between women and men with respect to the fact that as hypertensive patients, they are at higher risk of experiencing complications in case of COVID-19 infection (86.2 vs. 86.5%, respectively). But again, when comparing themselves to the general population, only 48.1% (*n* = 125) of the participants self-identified as at higher risk for complications due to COVID-19 (see Table 2).

In this study, only 15 participants (5.8%) reported having been tested for COVID-19 with real-time PCR (RT-PCR) and only 1 participant received a positive result (incidence of 3.84 per 1,000 inhabitants). However, 23.5% (*n* = 61) of the participants had the so-called “rapid test” or antibody test, with 3 showing a positive result. Both the tests were performed on 36 people (13.8%).

Knowledge about controlling the spread of the disease revealed 71.2% (*n* = 185) of the participants reported that handwashing should last at least 20 s, while 20% (*n* = 52) of the participants believed that the amount of time spent in handwashing did not matter, with no difference between genders (see Table 3). In addition, during quarantine, 87.7% (*n* = 228) of the participants reported leaving home at least once weekly and men did so more than 3 times weekly, significantly more than women did (refer to Table 3).

**Table 3:** Behaviors against COVID-19 risk of infection in a semi-urban population from Ecuador.

	**Men**	**Women**	***p* value**
	**(*n* = 74, 28.5%)**	**(*n* = 186, 71.5%)**	
Hand washing			
10 sec	5 (6.8)	17 (9.1)	0.8799
20 sec	52 (70.3)	133 (71.5)	
Does not matter	17 (23.0)	36 (19.4)	
Left home (times/week)			
Never	6 (8.1)	26 (14.0)	0.2170
Once	14 (18.9)	49 (26.3)	0.2617
2–3	27 (36.5)	79 (42.5)	0.4039
More than 3	27 (36.5)	32 (17.2)	0.0016
Stays away from others at work? Yes	67 (90.5)	165 (88.7)	0.8254
Does isolation at home help? Yes	58 (78.4)	153 (82.3)	0.4851
Stop using protective measures			
After a month	2 (2.7)	4 (2.2)	1.0000
After a year	12 (16.2)	29 (15.6)	1.0000
Until a vaccine is available	51 (68.9)	139 (74.7)	0.3553
Never	9 (12.2)	14 (7.5)	0.2352

**Figure 1:**
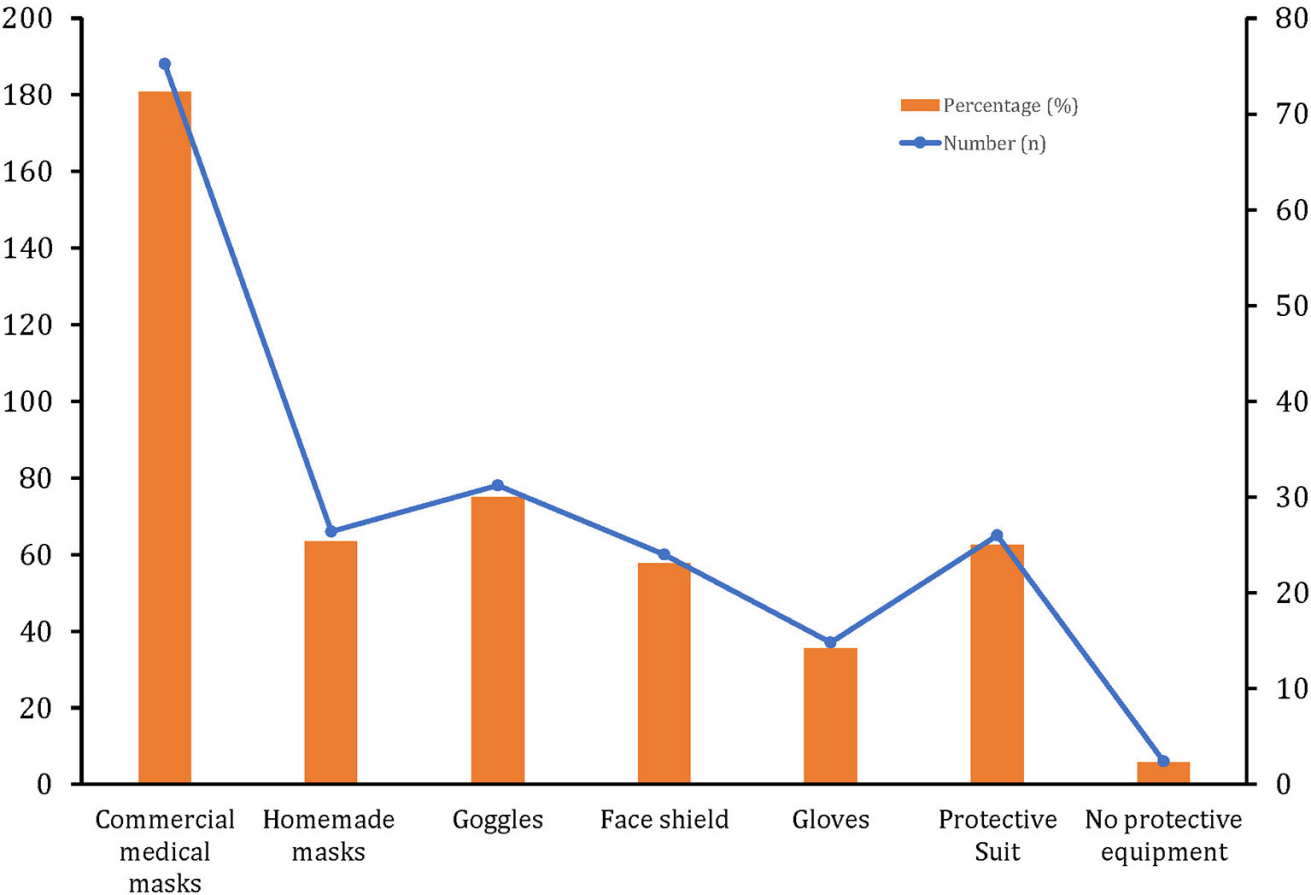
Personal protective equipment used by hypertensive people in a semi-urban population of Ecuador.

Almost all the participants (97.7%) reported the use of face masks, 25.4% (*n* = 66) of the participants wear homemade masks, while 72.3% (*n* = 188) of the participants purchased commercial ones. There was also other personal protection equipment reported by participants as shown in Figure 1.

For 89.2% (*n* = 232) of the participants, staying away from people who did not live in their household (social distancing) was useful protection against COVID-19 infection (Table 3). Similarly, 81.2% (*n* = 211) of people around the world believe that home isolation can be an appropriate measure to prevent COVID-19 infection.

**Figure 2:**
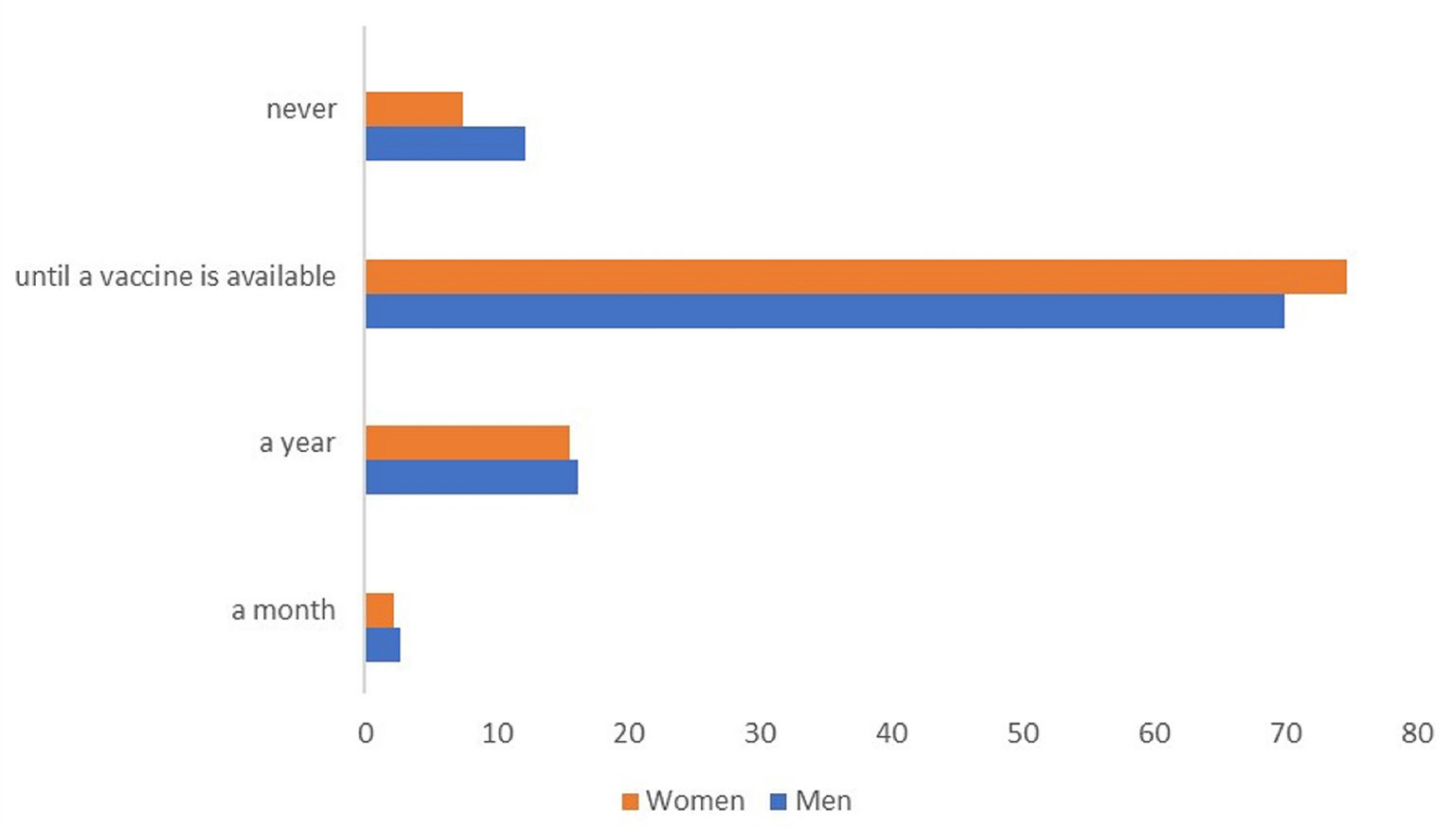
Perception about the duration of the use of preventive measurements against coronavirus disease 2019 (COVID-19) infection by hypertensive people in a semi-urban population of Ecuador.

Of the respondents, 73.1% (*n* = 190) participants believed that the use of protective measures should continue until a vaccine is available, while 15.8% (*n* = 41) of the participants believed that it should be a year (Figure 2).

## Discussion

All the subjects, in this study, knew that COVID-19 is caused by SARS-CoV-2, a very high degree of scientific literacy by a lay nonprofessional population, but not surprising as during the lockdown it was the key message provided permanently by all the media, as pilar to fight infodemic (16). In this study, although COVID-19 was recognized to be different than the common flu by almost 60% of the participants and 3 quarters of the sample were concerned about becoming infected in the nearest future, i.e., the next 2 or 3 months, less than half of those surveyed considered themselves at high risk of infection compared to the general population. A similar finding was reported in the United States at the beginning of the pandemic when people older than 60 years believed that they had a low chance of being infected (17).

In the hypothetical case of infection, most of these semi-urban people with hypertension considered themselves at high risk of presenting complications. However, once again, only 1 out of 4 identified their chronic disease as a risk factor for complications from COVID-19. This might be explained by the fact that more than 80% of these subjects were being treated for hypertension and more than 90% of these subjects were confident that their hypertension was well controlled. This perceived level of risk can be compared to studies during the H1N1 pandemic; wherein the general population, the perceived likelihood of infection was between 2 and 3 (1: not likely and 5: very likely), a number that was lower in people with better, self-reported health status (18).

It is important to note that our data collection was conducted between the 5th and 9th month of the pandemic (with the starting point considered to be the first lockdown), when substantial information about COVID-19 through the media, either correct or false, had been released, making people more aware of any repercussions or hyperalert with regard to COVID-19.

Thus, it is not surprising that almost all the participants in this study believe that handwashing was essential, although only 71% knew the appropriate length of time it should be carried out. However, almost all the participants in this study left their homes at least once a week, yet more than 80% thought that it was useful to practice isolation in their houses, away from others to avoid the infection.

This behavior agrees with a US study reporting that 70% of people adopted social distancing measures, but surprisingly younger people engage more in this practice than the older population (17). A study showed that people who have a greater perceived risk avoid public places, restaurants, shops, or travel (19). They also report a greater intention to comply with quarantine restrictions and avoid public transportation (20). This data supports our findings that high self-perceived risk in acquiring the disease makes people exhibit better behaviors.

In comparing people with high risk due to the severity of the disease and preventive behavior such as using a face mask (20), this study revealed that almost all the participants used protective measures such as surgical face masks alone or in combination with other types of face masks. Indeed, in Nigeria, adequate knowledge of COVID-19 was linked to greater participation in precautionary behavior based on the perceived risk by women but not men. It was also found that awareness campaigns and psychological intervention strategies on COVID-19-related activities may be particularly important for men more than women (21).

It is also known from studies on disease due to SARS that general knowledge of the causative agents of the diseases, the symptoms, their similarity to other diseases, and perceived risk of COVID-19 were associated with precautionary behavior in the population. Without a proven and acceptable pharmaceutical cure and in the face of the delay in the acquisition of vaccines worldwide, the best way to stop COVID-19 and prevent it from spreading may be to adopt precautionary behaviors and biosafety measures (22).

However, it has been reported that more than 80% of people used a face mask when in the grocery store, but only half used it when visiting friends and family. The use of face masks was predominantly among women, older people, the black and Hispanic population, and respondents with lower income. It was also more frequent in large urban areas (23). In this study, even when we can see that this evidence supports our finding that higher rates of women and Hispanics use face masks, we cannot be certain as to whether mask-wearing occurred in the setting of visiting family and friends or just for other errands.

Surprisingly, almost half of the participants in this study had been tested for COVID-19, although only 6% of the participants had a positive result confirmed, a finding that can be translated as an important self-perception of having been exposed to COVID-19 and potentially infected. This result could also mean that although most patients reported using personal protective measures when they left their houses and at the same time, the majority only left their houses once a week, they are concerned that the spread of the disease is coming from another source such as gatherings with family or friends without adequate protective measures being taken. This last observation is probably where future studies and prevention strategies should focus.

Finally, 73% of the participants mentioned that the use of personal protective equipment will end once there is a vaccine. However, this revealed a lack of adequate communication about the rationale for vaccination and its slow and progressive impact on the pandemic behavior. For example, in the particular case of Ecuador, an early SIR model showed that herd immunity will require vaccination of at least 55% of the population (24). However, in the early months of the COVID-19 pandemic, a global survey about optimism over the COVID-19 outbreak coming to an end showed that European and Asian countries had a more negative view of the situation and became even more pessimistic as time passed, compared to countries such as Brazil and Mexico that had a more optimistic view about the situation (25).

We recognize that the main limitation of this study was that it was conducted in a single health center in a specific community. Thus, the generalizability of this study is very limited. The findings are very limited to a very specific population. Also, most of the participants were women, although worldwide, it has been shown that men and women have an equivalent risk of infection (26). However, our results are particularly important because they refer to a vulnerable population and whose beliefs might be useful for avoiding the disease and its complications. These are a clear representation of the areas on which our efforts should focus during the COVID-19 pandemic. Even though most of the population understands the situation and the actions they should take, the concern is with the subgroup that does not understand COVID-19 and does not engage in this appropriate protective behavior. Care of this group is especially important due to its characteristics and difficulties with respect to the disease, but also because its members could potentially spread COVID-19 to other people. A great approach, not only for this group of people, should focus on information, education, and resources about COVID-19 and its possible repercussions.

## Data Availability Statement

The raw data supporting the conclusions of this article will be made available by the authors, without undue reservation.

## Ethics Statement

The studies involving human participants were reviewed and approved by Comite de Etica de Investigacion en Seres Humanos (2020-047M) - Universidad San Francisco de Quito. The patients/participants provided their verbal informed consent to participate in this study.

## Author Contributions

TA, IP, and ET conceptualized the study. TA, MC, DV, and MV collected and analyzed data. All the authors contributed to writing the initial draft and all the authors reviewed and approved the final version of the manuscript.

## Conflict of Interest

The authors declare that the research was conducted in the absence of any commercial or financial relationships that could be construed as a potential conflict of interest.

## Publisher's Note

All claims expressed in this article are solely those of the authors and do not necessarily represent those of their affiliated organizations, or those of the publisher, the editors and the reviewers. Any product that may be evaluated in this article, or claim that may be made by its manufacturer, is not guaranteed or endorsed by the publisher.
