# Supplementary material for: Risk Perception and Knowledge, Attitudes, and Practices Against COVID-19 in a Hypertensive Population From a Semi-Urban City of Ecuador
Source: Front Public Health. 2021 Dec 14;9:734065. doi: 10.3389/fpubh.2021.734065 (PMC8712554; doi:10.3389/fpubh.2021.734065)
Supplement: Supplementary file 1 [file Data_Sheet_1.PDF]

1. Age

2. Gender
    a. Female
    b. Male

3. Do you receive any drugs to control your blood pressure?
    a. Yes
    b. No

4. Do you think your blood pressure is under control with this treatment?
    a. Yes
    b. No

5. Have you been diagnosed with any of the following diseases in addition to hypertension? (more than one is allowed)
    a. Diabetes
    b. Renal disease
    c. Cancer
    d. Thyroid disease
    e. Obesity
    f. Hearth disease
    g. Lung disease (COPD)
    h. CNS Diseases (depression, dementia, stroke, Parkinson´s disease)
    i. None

6. Do you receive any medication for that (those) disease(s) (linked to #5)?
    a. Yes
    b. No
    c. N/A

7. Do you think that (those) disease(s) is (are) under control with this (these) treatment(s) (linked to #5)?
    a. Yes
    b. No
    c. N/A

8. Do you know about the COVID-19 or the coronavirus?
    a. Yes
    b. No

9. Do you think that the disease due to the COVID-19 is similar to the common flu?
    a. Yes
    b. No

10. Do you think that your risk of getting infected with the COVID-19 compared to the general population is?
    a. Lower
    b. Same
    c. Higher

11. Do you think that your risk of getting infected with COVID-19 is?
    a. Very low
    b. Low
    c. High
    d. Very high

12. In case you are infected with COVID-19, do you think your risk of complications compared to the general population will be?
    a. lower
    b. same
    c. higher

13. In case you are infected with COVID-19, do you think your risk of complications will be?
    a. Very low
    b. Low
    c. High
    d. Very high

14. Have you been ever tested against COVID-19?
    a. Rapid test (antibodies)
    b. RT-PCR
    c. Tested but no idea which test
    d. Never tested

15. How was the result of your test (linked to #14)?
    a. Positive
    b. Negative

16. How long do you wash your hands (mínimum)?
    a. 10 sec
    b. 20 sec
    c. It does not matter while you wash your hands
    d. It is not important to wash your hands

17. Once you leave home, what kind of protection do you use?
    a. Commercial medical mask
    b. Homemade mask
    c. Goggles
    d. Face protector
    e. Gloves
    f. Protective suit
    g. None

18. Do you think that staying away, at least a meter, from other people is effective to avoid the transmission of COVID-19?
    a. Yes
    b. No

19. Do you think that staying at home is effective to avoid the transmission of COVID-19?
    a. Yes
    b. No

20. How long do you think that personal protective measurements will be in place?
    a. A week
    b. A month
    c. A year
    d. Until a vaccine or treatment is available
    e. Never will leave there

21. In the last month, how many times did you leave home?
    a. Once weekly
    b. 2-3 times weekly
    c. 4-5 times weekly
    d. >5 times weekly
    e. Never leave home
